# BS-QUAL: Measuring student perceptions of service quality in business schools, an exploratory study

**DOI:** 10.1007/s11233-023-09118-x

**Published:** 2023-02-13

**Authors:** Llorenç Bagur-Femenias, Josep Llach, Marian Buil

**Affiliations:** 1 UPF Barcelona School of Management, Barcelona, Spain; 2 Escola Politècnica Superior, Universitat de Girona, Girona, Spain; 3 Escola de Ciències Socials i de l’Empresa Tecnocampus – UPF, Mataró, Spain

**Keywords:** Service quality assurance, Business schools, Business education, Student satisfaction, Excellence in higher education, Perceived quality

## Abstract

The number of Business Schools (BS) and their market share had increased in the last decades. Positioning strategy of BS is crucial in today’s competitive and changing environments. Thus, paying attention to student’s satisfaction and the factors, which motivate their selection, are relevant for service quality assurance in BS. This paper designs a scale to measure these perceptions as a useful tool for BS managers in the pursuit of excellence. Using a mixed analysis methodology, the most prominent dimensions detected in the literature were validated by BS managers and later by BS graduates. Internal and external dimensions compose the resulting scale, named BS-QUAL. The internal dimensions are related to Academic staff, Services, Facilities, and Sustainability while the external factors are related to Preparation for the future, Internationalization and Notoriety. BS-QUAL could be useful for the top management of BS to develop strategies that minimize the distance between student expectations and service provided.

## Introduction

Different studies, based on different sources of information, have attempted to quantify the number of business schools that operate worldwide (Peters, 2007). Most agree that their numbers are high and increasing. Certain authors, such as de Freitas et al. (2016), even place their numbers at above 20,000. The struggle to position themselves in the market is fierce, and it is of vital importance to be competitive and able to adapt to new environments and the real needs of organizations (Starkey and Thomas, 2019).

There used to be two main strategies a business school could choose to position itself in the market (Thomas, 2007; Trim, 1994). The first of these, the most global, involved gaining notoriety through a presence in the main rankings and by obtaining quality accreditations worldwide, the so-called triple crown. However, the numbers confirm that achieving results with this strategy is not easy at all. According to the numbers provided by de Freitas et al. (2016) on the quantity of business schools, fewer than 1% manage to join such a select group.

Under the second strategy, which is less global and more regional, a business school opted to position itself as a reference in a given market segment within its area of influence. Segmentation by sector, by being a leader in quality/price, by specialization in methodology (online, for example), or by a high degree of quality labour insertion, was and still is a common means to position a brand.

However, the times of stability for the business school have ended. The arrival of the era of disruption and of constant changes in demands and needs necessitate a strategic review aimed at the continuous observation of the market and its agents (Thomas, 2009). Many authors have argued that, in general, the quality of the training provided by a business school does not meet the expectations of students or employers (Bennis and O’Toole, 2005). The general criticism tends to focus on the fact that business schools do not provide students with the competencies and skills necessary to enter the labour market. Additionally, criticism focuses on their absence of humanistic, ethical, or sustainability training, which does not reflect in reality how business is conducted and which results from an excessive look “inward” that causes business schools to lose sight of key agents such as students and employers and their perceptions.

In this sense, the present study aims to shed light on the perspectives of students and, specifically, on how they perceive the quality of the training received in business school. In line with the Theory of Planned Behaviour (TPB) (Ajzen, 1985), students’ perceptions will be influenced by taking into consideration the influence of personal evaluations, perceived social pressure, and perceived control in predicting the intention to perform a given behaviour related to business schools. The design of a scale to measure these perceptions can be useful for business school managers in their search for excellence.

This article is divided into six clearly differentiated sections as follows. First, this introduction highlights the importance of the topic of study and its objectives. The second section analyses the previous literature. A third section explains the methods used to achieve our objectives. A fourth section details the design of a business school perceived quality scale, and in two final sections, the results are analysed and conclusions are detailed.

## Literature review

Defining human behaviour is a difficult task given the complexity of the term. The Theory of Reasoned Action (TRA) (Ajzen & Fishbein, 1980) and Theory of Planned Behaviour (TPB) (Ajzen, 1985) try to explain the behaviour of human beings.

The TPB (Ajzen, 1985, 1987, 1991) is used to try to predict behaviour. It proposes four factors in a mediation model. The first component is the attitude about a behaviour and what one thinks about something or a new habit or purpose. The second element is social norms, indicating the opinion that people close to us have about something we are going to do. Social norms demonstrate that the environment influences what we do. The third factor is perceived behavioural control or the capacity we have to intervene in what we do. The last component is the intention to perform a behaviour, which Azjen determines as the most important. This intention is more relevant than the rest of the elements and even than what one thinks. People who do not perceive themselves to have control over what they do will have less intention to perform a behaviour and will directly influence the behaviour and its effectiveness. Furthermore, Ajzen and Fishbein (1980) suggest that an attitude towards something is determined by one's salient beliefs about that thing and the affective aspect related to those salient beliefs.

The TPB has been used in a variety of research papers. Young et al. (1991) describe the operationalization of the TPB with examples from two studies of bovine spongiform encephalopathy behaviour in older women. Ajzen and Driver (1992) applied the theory to predict the leisure intentions and behaviours of college students. The survey proposed measures attitudes, subjective norms, moods, involvement, perceived behavioural control and intentions regarding five leisure activities. Shih and Fang (2005) present a new way of gauging customer loyalty and predicting their possibility of defection with reference to a set of quality attributes of satisfaction and three types of belief using the theory of planned behaviour (TPB). Yakasai and Jusoh (2015) investigate the factors that influence the use of digital coupons among university students in Kuala Lumpur. The results show that attitudes are the strongest predictor, followed by subjective norms and perceived behavioural control. Sultan et al. (2020) examined the moderating effects of perceived communication, satisfaction and trust on the intention behaviour gap and the perceived behavioural control (PBC)-behaviour gap in the theory of planned behaviour (TPB) model using the partial least squares-based structural equation modelling (PLS-SEM) technique. The findings confirm that perceived communication, satisfaction and trust positively and significantly enhance purchase behaviour and lessen gaps in the intention behaviour and PBC-behaviour relationships in the TPB model. Prasetyo et al. (2021) determine the factors influencing customer satisfaction with and loyalty to an online food delivery service (OFDS) during the new normal of the COVID-19 pandemic in Indonesia utilizing the extended theory of planned behaviour and conducting structural equation modelling (SEM). Luceri et al. (2022) used the TPB to determine factors underlying mobile shopping behaviour. They provide and test a comprehensive framework for the key drivers of consumers’ initial adoption and the continuance intention to use mobile devices for purchases.

There are other studies that identify variables and dimensions that affect perceptions of quality of higher education students and their general satisfaction, especially towards business schools. As described by Urgel (2007) and Marimon et al. (2019), many studies have focused on these variables, as the quality service of academic institutions has become a crucial topic influencing their students (Buela-Casal et al., 2013). Nevertheless, the research on student perceptions of academic quality is insufficient and requires further investigation to define which components of university education shape perceptions of quality among students (Sokoli et al., 2018). As Parameswaran and Glowacka (1995) demonstrate, student satisfaction is the only indicator of the performance of quality service for suppliers of education, and it is important to give priority to students as customers of education as well as meet their expectations (Peng et al., 2012). In contrast, some authors (McMahon, 1992; Mazzarol, 1998) point out that the quality of education relies on stakeholders who interact with services delivered by higher education institutions. However, as noted by Mai (2005), analysing public and private universities or business schools is not the same. If to this last factor we add that it is a sector in continuous evolution (Starkey & Thomas, 2019) and in which, in a few years, the literature will be outdated because of the disruptive stage we are experiencing in the higher education sector (Barrett et al., 2019), we have chosen to review the literature of relevant studies from 1990. Additionally, and in line with the framework provided by Bagur-Femenias et al. (2020), although showing some slight differences, the variables were grouped into two large groups of dimensions that affect the perception of the quality of service among students. These are dimensions of internal and external factors. In this literature review, we dedicate one section to each group. Furthermore, following Ajzen & Fishbein’s TPB, the dimensions are represented as important salient beliefs related to one's attitude towards satisfaction with the BS experience.

### Dimensions related to internal factors

In this section, we pay special attention to dimensions related to variables that directly depend on internal decisions made by the top management of business schools and in which third parties do not intervene. For this group of variables, we find mainly what Borden (1995) called academic factors as well as the so-called tangible factors. We must understand these as facilities, means, or services made available to the student (Oldfield & Baron, 2012). We must realize then that when we talk about internal factors, we are referring not only to academic decisions but also to investment decisions and the placement of resources that aim to make available to the student not only the best programme but also the best environment in which to study it. In other words, higher education institutions need to consider both personal and institutional factors affecting students’ satisfaction (Soutar & Turner, 2012; Price et al., 2013). Srikatanyoo and Gnoth (2012) defined personal factors as age, gender, temperament, grade point average (GPA), preferred styles of learning and employment status.

The number of articles in terms of academic dimensions shows evidence that this is one of the most studied internal satisfaction factors. These aspects include aspects such as the qualifications, aptitudes, and comportment of teachers and aspects related to courses, including their usefulness and content and the number of students per class (Borden, 1995; Leblanc & Nguyen, 1997 or Gibson, 2010 among others). These institutional factors include instruction quality, promptness, quality, feedback and the clarity of expectations, styles of teaching, class size and institution research (Sadiq Sohail & Shaikh, 2014). Furthermore, as Owlia and Aspinwall (1996) pointed out, the satisfaction of professors makes an important contribution to the authenticity and accuracy of provided services of higher education.

Other internal factors in addition to what happens strictly in the classroom include the so-called student journey. This entails not only a course itself but also aspects before and accessory to the course. Management personnel have an essential role in the student journey. From a highly informative process before enrolment to good administrative support during the course, key aspects of perceptions of the quality of service are considered by authors such as Borden (1995), Elliot and Shin (2002), De Shields et al. (2005), and Gibson (2010).

Browne et al. (1998) were among the first to introduce personal development factors as key variables for satisfying students. The skills developed from the programme studied become part of the student's curriculum and are considered more valuable. The promotion in the classroom of managerial skills, a critical spirit, and simple intellectual growth become central aspects of higher education. At the end of the twentieth century and beginning of the twenty-first century, the paradigm in business school programmes changed: What is important is more than what is learned; schools have shifted from the knowledge age to the knowledge-and-skills age.

Similar to the developed skills, the values the students acquire have changed. It is no longer enough to acquire knowledge and skills, as business schools must educate and set an example in terms of sustainability. Many authors highlight this type of variable. The contribution to the business school community of having clear environmental, ethical, or gender policies has gone from being peripheral to being central in the strategy of a business school (Dyllick, 2015). Similarly, Sokoli et al. (2018) found that the level of undergraduate satisfaction correlates with students’ ability to access enough resources to satisfy social and academic interests. The authors conclude that the social experience and academic sentiments within higher education institutions improve the overall campus experience.

Another relevant dimension related to internal factors has to do with innovation. In times of notable technological change, being up to date with learning methodologies, technological media and online platforms, and content is key to the student's perception of quality. Gibson (2010), Dyllick (2015) and Lagrosen (2017) put teaching at the centre of the equation.

Finally, a last relevant dimension of internal factors is where the course is given: the material means available. This dimension includes variables related to the building in which the business school is located and the services and facilities provided by academic institutions (Oldfield & Baron, 2012) that emanate from it, such as the presence of a restaurant, study rooms, and libraries. It also includes the environment and amenities that the location provides, the ease of parking, good public transport connections, campus security, and the cleanliness or image projected by the building. This is one of the groups of variables that authors have raised the most. Standing out among these authors, we must cite Leblanc and Nguyen (1997), who attribute what they call “physical aspects” of quality to the variables that students see as having “functional value”. Borden (1995), Delaney (2001), and Gibson (2010) echo these thoughts in different studies carried out with different methods whereby these variables are, without a doubt, key to student satisfaction.

### Dimensions related to external factors

Leblanc and Nguyen (1997) analyse student satisfaction by breaking it down into seven different “impacts” experienced by the student that increase the perceived quality of services received. Most of them have to do with internal variables described in the previous section; however, "name value" or reputation, "emotional value" or belonging, and being part of "functional value" after graduation are some of what we call external factors.

Behind the institutional reputation factor, there is clear confusion over its definition and conceptualization, as Sokoli et al. (2018) pointed out. Some authors (Buela-Casal et al., 2013; Tam, 2013) associate reputation with the name and profile of the organization; others (Srikatanyoo & Gnoth, 2012) associate it with the image of the organization. Krampf and Heinlein (2014) agree on a definition of reputation as a view of provided services that is cognitive and relatively communicative and that is affected by tangible and intangible elements, communication and values (Price et al., 2013). Furthermore, reputation acts as a driving force behind students’ acceptance and satisfaction with a chosen university (Veloutsou et al., 2014).

The presence of a school in rankings or its brand projection and implications are key variables among external factors. Jewett (2012) even concludes that all the factors that imply notoriety and that project a positive perception of a business school are even more relevant than academic topics in the selection of a programme. As a counterbalance to Jewett (2012), Lagrosen (2017) states that prestigious external accreditations, such as AMBA, EQUIS, and AACSB, are important and highly valued assets by students. However, he believes that in some cases, these quality certification models focus more on outputs than on people.

Urgel (2007) provides variables that we categorize under knowledge transfer. A business school must transmit value to society, organizations, and individuals. The most common ways of transferring knowledge have to do with being in continuous contact with the environment and analysing and updating it. In this sense, being part of relevant research projects; having a continuous relationship with companies through professorships, consultancies, or agreements; or conducting relevant studies with an impact on the press improves a student's perception of a business school.

In an increasingly globalized world, the international dimension is becoming increasingly important. Alves and Raposo (2007) highlighted the importance of the international presence and notoriety of a business school for students. The student as more valuable perceives variables such as student mobility through agreements with other prestigious international business schools and the school’s presence in international projects, increasing perceptions of quality.

Finally, yet importantly, we find variables related to the preparation of the student for the future. These variables are the outputs that show their worth after the end of a programme. Most of these variables are oriented towards the integration of the student in the labour market and the improvements that the integration of a programme in a business school entail. Many quality assurance systems, including those of the most famous international accreditations and rankings, outweigh these variables (Lagrosen, 2017). These rankings consider, for example, the salary increase after the completion of a programme and the rate at which graduates land quality jobs as clear indicators of programme quality, as well as students being prepared to integrate into the labour market and the recognition by employers of graduates’ skills.

**Fig. 1:**
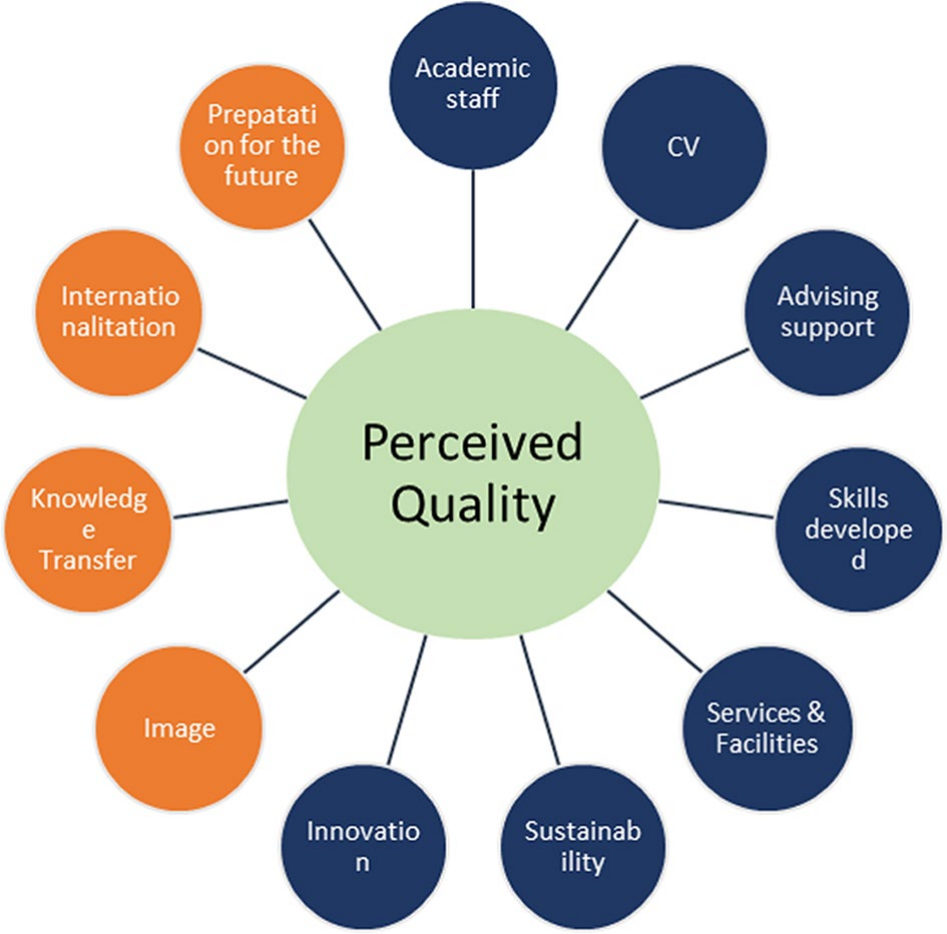
Hypothesis derived from the literature review. Source: own elaboration

Based on the above literature review, the objective of this study is twofold. First, we analyse whether the quality of service perceived by a business school student is a multidimensional construct (see Fig. 1). Second, through empirical analysis, we design and validate a scale that allows for the evaluation of the quality of services provided by a business school.

## Methods and sample

The above analysis of the literature shows that many studies have analysed the effects of different variables, or groups of independent variables, on the quality perceived by the student of a higher education programme. However, evidence of recent studies that have attempted to design and validate a scale that integrates all the variables described in the literature review has not been found. Churchill (1979) provided scientific guidelines on how to design and validate this type of questionnaire, although since 1979, the structure and method proposed by the author has evolved.

In our specific case, and with the objective of establishing a scale to evaluate perceived quality and student satisfaction with a business school, an analysis with three clearly differentiated stages was designed:

- **Stage 1:** We first performed an exhaustive review of the literature with the aim of detecting the key variables that affect perception of quality and student satisfaction with business schools. In this first analysis, 66 variables cited by previous studies were detected.
- **Stage 2:** In Stage 2, a mixed analysis method was used, mixing purely quantitative techniques such as questionnaires with purely qualitative methods such as interviews to enrich the results. Specifically, the so-called mixed complementation method described by Bericat (1998), Callejo and Viedma (2005), Creswell and Plano Clark (2007), and Verd and López-Roldán (2008) was used.

With the initial questionnaire of 66 variables in hand, 10 top managers of business schools based in Barcelona were interviewed. The objective was to enrich the survey with possible variables that did not appear in the literature. In this round of interviews, seven new variables appeared that we needed to take into account and were added to the initial 66.

Once the final questionnaire with 71 items was written, the same 10 managers were asked to score, on a Likert scale from 1 to 5, the importance of each variable according to their criteria as professional experts in the area and establish a first screen. After analysing the results of the survey and following the method used by Marimon et al. (2019), 56 variables were incorporated into the final questionnaire to be validated by the students.

- **Stage 3:** In this last phase of the process, the final validation of the questionnaire was carried out. The final version was sent to recent graduates (in the last 5 years) of Spanish business schools. We obtained 272 valid responses, which were statistically analysed to define the final scale resulting from the present study. The questionnaire focused on people who had recently obtained their degrees because business schools are undergoing continuous change (Barrett et al., 2019). It was also considered necessary to focus only on people who had already completed their studies in business schools and to ignore those who were still in school. This decision was based on the need to evaluate the satisfaction of those who had already completed the "journey" of earning a master's degree: from being accepted to having completed their studies, joining the labour market, progressing in their professional careers, and using the school job bank.

The final questionnaire included, apart from the aforementioned variables, an additional section in which respondents were asked about sociodemographic information. All the items, with the exception of sociodemographic information, had to be completed by the respondent on a 5-point Likert scale where they indicated their degree of agreement/disagreement. Table 1 shows the sociodemographic characteristics of the sample in detail. Responses to the survey were obtained in the month of February 2020. The respondents were balanced in gender and age distributions. The majority of respondents were of Spanish nationality.

**Table 1:** Sociodemographic information

		Number	%
Gender	Male	130	47,8%
	Female	142	52,2%
	Total	272	100,0%
Age	< 25 years	6	2,2%
	Between 25 and 30 years	63	23,2%
	Between 31 and 35 years	75	27,6%
	Between 36 and 40 years	71	26,1%
	Between 41 and 45 years	50	18,4%
	> 46 years	7	2,6%
	Total	272	100,0%
Country	Spain	262	96,3%
	Europe	3	1,1%
	LATAM	6	2,2%
	Other	1	0,4%
Type of course	Official Master	194	71,3%
	Non Official Master	65	23,9%
	Other	13	4,8%
	Total	272	100,0%

## BS-QUAL scale proposal

In this section, we systematically analyse each of the three stages described in "Methods and sample" section of this article. Hereinafter, we call the scale defined in this article BS-QUAL, referring to the final objective of this study: to measure the respondent’s perceived quality (QUAL) of a programme offered by a business school (BS).

### Stage 1: Initial grouping of variables into dimensions

Following the guidelines set by Bagur-Femenias et al. (2020) and to offer more detail and concreteness, we classified the 66 variables detected in the literature into 11 dimensions or large groups of items, expanding on the 10 original dimensions of the study. Specifically, more weight was given to the “knowledge transfer” dimension, separating it, per our hypothesis, from aspects related to the research, image, and notoriety of the business school.

### Stage 2: Focus group with business school managers

As mentioned in "Methods and sample" section and with the aim of enriching and/or resizing the constructs designed based on the literature, 10 interviews were held with current senior managers of business schools. During these interviews, seven new variables were incorporated (marked with an asterisk and in bold) into the initial 66: one in the Sustainability dimension, three in Knowledge Transfer dimension, one in the Innovation dimension, and two in the Preparation for the Future dimension. Appendix Table 7 shows the details and classifications of the 71 resulting items.

After the statistical analysis of the responses issued by business school managers, 15 of the 71 initial variables were eliminated from the questionnaire following the method used by Marimon et al. (2019), all with mean Likert scores of below 3.5 and/or significantly high variances.

### Stage 3: Analysis of the responses of alumni to define the scale.

To avoid conditioning the dimensions, an exploratory factor analysis (EFA) was performed on the entire sample. From this analysis, six dimensions emerged, and the variables were mostly grouped naturally into dimensions already described in the literature. Note that of the 11 initial dimensions, five disappeared when we applied the required criteria of loading greater than 0.6 in the dimension itself, not loading more than 0.5 in another (in fact, in no case exceeded 0.4), and showing an item-to-total correlation of more than 0.5 (Bernardo et al., 2012; Ladhari, 2012; Wolfinbarger & Gilly, 2003).

Despite the above, and given that the variables AS1, SERV10m and IMG2 narrowly missed the 0.6 load but exceeded the 0.5 load required by other authors, we decided to keep them in the model while waiting for the results of the subsequent confirmatory study. Therefore, as a result of this analysis, the model was reduced to 23 variables grouped into six dimensions (see Table 2).

**Table 2:** Initial EFA

	Notoriety	Academic staff	Sustainability	Internationalization and Relations**	Services and Facilities	Preparation to the future
AS1	0.281	**0.566**	0.070	0.398	0.131	0.101
AS2	0.008	**0.809**	0.058	0.127	0.101	0.188
AS4	0.107	**0.731**	0.156	0.107	0.205	0.035
AS5	0.065	**0.745**	0.109	0.148	0.067	0.249
SERV2	0.038	0.107	0.162	0.178	**0.705**	0.198
SERV9	0.186	0.108	0.097	0.175	**0.713**	0.066
SERV10	0.295	0.222	0.358	0.082	**0.515**	-0.023
SERV11	0.070	0.127	0.200	0.096	**0.700**	0.138
SUST2	0.216	0.309	**0.659**	-0.089	0.193	0.070
SUST3	0.137	-0.048	**0.739**	0.222	0.218	0.148
SUST5	-0.001	0.055	**0.842**	0.127	0.148	0.058
SUST6	0.142	0.207	**0.644**	0.342	0.136	0.131
IMG2	0.490	0.086	0.244	**0.585**	0.042	0.177
IMG4	0.199	0.168	0.154	**0.763**	0.176	0.107
INT1	0.368	0.236	0.108	**0.593**	0.265	0.150
INT2	0.065	0.180	0.139	**0.726**	0.182	0.151
KT5	**0.720**	0.025	0.222	0.094	0.284	0.047
KT6	**0.658**	-0.047	0.246	0.124	0.269	-0.012
IMG5	**0.753**	0.151	0.031	0.283	-0.006	0.207
IMG6	**0.716**	0.314	-0.097	0.169	-0.021	0.322
FUT1	0.193	0.246	0.033	0.116	0.221	**0.757**
FUT2	0.358	0.056	0.224	0.086	0.189	**0.679**
FUT5	-0.003	0.233	0.124	0.228	0.048	**0.740**

In bold, the items of each dimension

In Table 2, two hybrid dimensions are included but were not considered in the initial model derived from the literature despite being completely congruent and easily explained. First, a dimension was created from two variables of the “knowledge transfer” dimension, the school’s impact on the press and its impact on social networks, and from two variables of the “Image” dimension, its presence in rankings and its market share. These items are clearly related to the global visibility of the institution, so the new construct was renamed “Notoriety”. In a similar way, a dimension was created from items of "Internationalization", such as those related to the agreements signed by the institution with other centres and two variables of the "Image" dimension: being internationally accredited and being part of a prestigious network of schools of business. Clearly, all of these items refer to how the business school interacts externally with external accrediting agents and with other business schools. In this sense, it was optimal and descriptive to name the new dimension “Internationalization and relations”.

With the six definitive dimensions, six new EFAs were performed, launching each dimension independently to contrast the reliability and validity of each construct. As shown in Table 3, the three variables conserved in the model, despite not meeting the minimum load requirement in the previous stage, exceed the minimum required in this phase of the analysis, having a dimension loading value of above 0.7 in all cases and therefore complying with the permanence requirements of the item in the construct.

**Table 3:** EFAs and reliability analysis

	Academic Staff	load	Services and Facilities	load	Sustainability	load	Internationalization and Relations	load	Notoriety	load	Preparation to the future	load
	AS1	0.738	SERV2	0.750	SUST2	0.728	IMG2	0.800	KT5	0.787	FUT1	0.849
	AS2	0.831	SERV9	0.766	SUST3	0.812	IMG4	0.821	KT6	0.732	FUT2	0.812
	AS4	0.775	SERV10	0.738	SUST5	0.841	INT1	0.822	IMG5	0.828	FUT5	0.784
	AS5	0.807	SERV11	0.740	SUST6	0.788	INT2	0.777	IMG6	0.789		
% explained variance	34.837		8.780		7.575		5.015		4.858		4.578	
Cronbach’s Alpha	0.793		0.737		0.798		0.819		0.789		0.747	
Composite Reliability	0.867		0.835		0.871		0.880		0.864		0.856	
Average Variance Extracted	0.621		0.560		0.629		0.648		0.615		0.664	

Regarding the indicators of the internal consistency of the dimensions, as shown in Table 3, the minimum Cronbach’s alpha of 0.7 (Nunnally & Bernstein, 1994) is far exceeded in all cases, such as in composite reliability. Additionally, the average variance extracted (AVE) shows optimal values in each dimension, far exceeding the target of 0.5 required by the construct of Fornell and Larcker (1981).

Table 4 confirms the discriminant validity of the model. Note that, in all cases, the square root of the AVE (in bold on the diagonal) is greater than the correlation of each dimension with the others (Fornell & Larcker, 1981).

**Table 4:** Discriminant validity analysis

	1	2	3	4	5	6
Academic Staff	***0.788***					
Services and Facilities	0.429^**^	***0.748***				
Sustainability	0.377^**^	0.549^**^	***0.793***			
Internationalization and Relations	0.518^**^	0.510^**^	0.487^**^	***0.805***		
Notoriety	0.387^**^	0.435^**^	0.378^**^	0.606^**^	***0.784***	
Preparation to the future	0.490^**^	0.430^**^	0.387^**^	0.504^**^	0.479^**^	**0.815**

In the diagonal, the square root of AVE in bold

**Significant at 0.01 (bilateral)

The last step necessary to validate the six dimensions resulting from the EFA involved performing confirmatory factor analysis (CFA). The results obtained are highly satisfactory, confirming the robustness of the dimensions both in load per item (see Table 5) and in relation to fit indices.

**Table 5:** Confirmatory factor analysis and fit indices of the model

Dimension	Items	Load	t-value	r2
Academic Staff	AS1	0.674	-	0.454
	AS2	0.745	9.973	0.555
	AS4	0.672	9.234	0.452
	AS5	0.732	9.851	0.535
Services and Facilities	SERV2	0.637	-	0.405
	SERV9	0.642	8.284	0.412
	SERV10	0.671	8.546	0.450
	SERV11	0.618	8.059	0.383
Sustainability	SUST2	0.616	-	0.379
	SUST3	0.756	9.359	0.572
	SUST5	0.744	9.271	0.554
	SUST6	0.731	9.168	0.535
Internationalization and Relations	IMG2	0.751	-	0.565
	IMG4	0.729	11.502	0.531
	INT1	0.779	12.293	0.607
	INT2	0.645	10.134	0.415
Notoriety	KT5	0.647	-	0.418
	KT6	0.574	8.209	0.330
	IMG5	0.802	10.332	0.664
	IMG6	0.757	9.973	0.573
Preparation to the future	FUT1	0.768	-	0.591
	FUT2	0.728	10.388	0.529
	FUT5	0.623	9.129	0.388
Fit indices of the model				
Satorra-Bentler χ2		400.252		
Degrees of freedom		215		
χ2 / DF		1.862	< 5	Wheaton et al. (1977)
Comparative Fit Index (CFI)		0.883	Close to 0.9	Hu and Bentler (1999)
RMSEA		0.072	< 0.1	MacCallum et al. (1996)
GFI		0.855	> 0.9	Byrne (1994)
AGFI		0.814	> 0.8	Byrne (1994)

The fit indices presented in the lower part of Table 5 were calculated using EQS 6.0 with a maximum-likelihood configuration. They show that the goodness of fit of the model due to more than three statistics fulfils the values recommended in the literature (Schermelleh-Enge et al., 2003).

To conclude this section, the analysis began with 71 variables collected from the analysis of the literature, grouped into 11 dimensions. After the focus group with business school managers and after the entire validation process employing the responses of alumni of business schools, the scale was reduced to six key dimensions formed by 23 variables. Table 6 systematically shows the process by which the six final dimensions were reached.

**Table 6:** Step by step summary of the analysis performed

Original Dimension	Literature Review	Focus Group	Exploratory Analysis	Definitive Dimension
Academic staff	7	6	4	Academic staff
Classes / CV	5	4	-	
Advising support	4	4	-	
Skills developed	5	5	-	
Services and facilities	12	6	4	Services and Facilities
Sustainability	8	7	4	Sustainability
Image	9	9	4	Notoriety
Knowledge transfer	7	4	-	
Internationalization	5	2	4	Internationalization and relations
Innovation	4	4	-	
Preparation to the future	5	5	3	Preparation to the future
Number of items remaining	71	56	23	

## Discussion of the results

This article presents a proposed scale (BS-QUAL) for assessing the quality of service of a business school as perceived by its students. The scale incorporates factors that include the entire process of what in the sector is known as the “student journey”, not only reflecting the impact of what happens in the classroom on the perception of quality but also the effect that certain indirect and external factors have on this satisfaction. To the best of our knowledge, this is the first study to establish a scale to measure the perceived quality of service at business schools using the method designed by Churchill (1979).

**Fig. 2:**
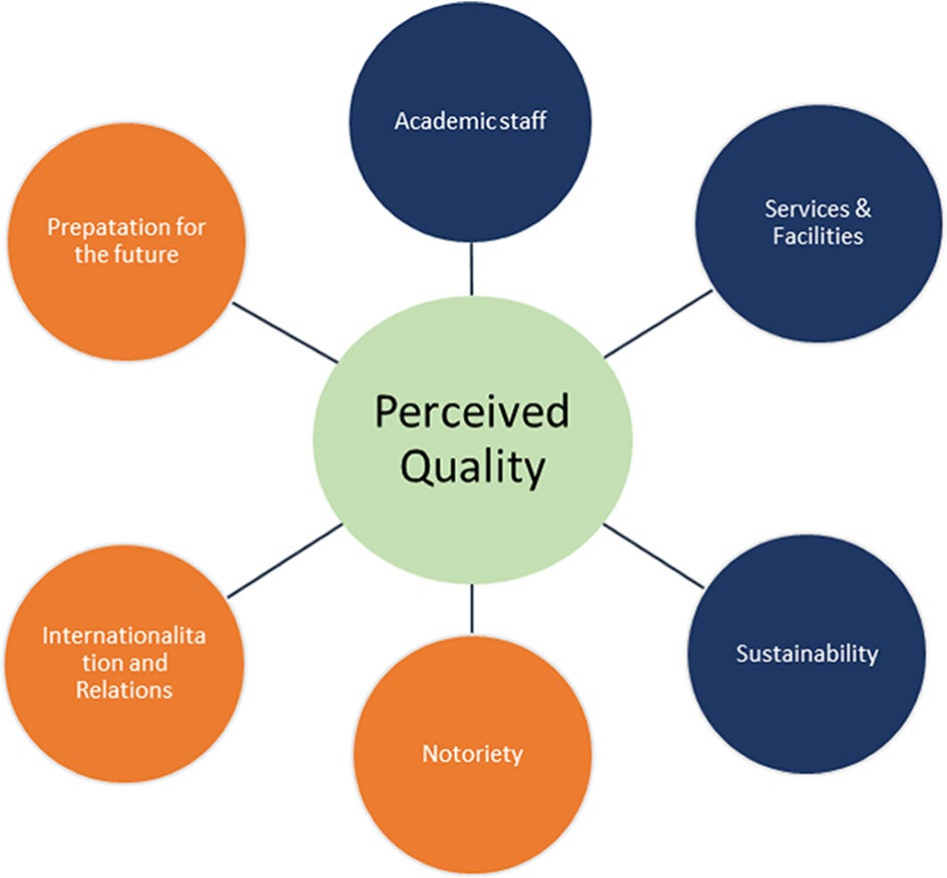
Graphical representation of the multidimensional construct “Perceived Quality”. Source: own elaboration

As one of the contributions of this article, it shows that the quality perceived by the graduate of a business school is the result of six dimensions (see Fig. 2), which could be classified into two large groups: internal and external factors. This classification is in line with the hypothesis proposed by Bagur-Femenías et al. (2020).

Internal factors are considered to be those the business school can directly influence. In our case, we discuss two dimensions: “Academic staff” and “Services and facilities”. Undoubtedly, the profile of the contracted faculty as well as the promotion of good practices in teaching and research are under the control of the business school. In the present study, in line with many previous studies (Borden, 1995), the profile, reputation, and skills of the teacher (Owlia & Aspinwall, 1996) were established as key to the satisfaction of the business school graduate. We should also mention the importance of what Parasuraman et al. (1988), in the SERVQUAL scale, called tangibles. In BS-QUAL, this dimension is renamed “Services and facilities” for a better description of the dimension.

It is noteworthy, however, that four dimensions disappeared that have been highly studied in the literature and that have to do with the aforementioned internal factors. These are “Classes/curriculum”, “Advising support”, “Innovation” and “Skills developed” (Borden, 1995; Browne et al., 1998; De Shields et al., 2005 or Gibson, 2010; Dyllick, 2015 or Lagrosen, 2017; among many others). Their exclusion seems to indicate that people who choose to attend a business school have a clearly results-oriented profile and seem to underweight the received value of variables of “accessory services” and that are not directly related to the main service, which seem to be, in terms of internal factors, the knowledge acquired and the tangible benefits received. The knowledge acquired is part of the academic objectives of students; for this reason, this result is in line with Sokoli et al. (2018), who stated that student satisfaction is focused on obtaining resources to expand social and academic objectives. The results obtained for the internal factors of knowledge acquired and the tangible benefits confirm that variables such as an adequate number of people in the classroom, time compatibility, administrative support received, and variables related to teaching innovation and acquired skills are not relevant to the final scale (see Appendix Table 8). These results run contrary to previous literature reviews (Borden, 1995; Browne et al., 1998; De Shields et al., 2005 or Gibson, 2010; Dyllick, 2015 or Lagrosen, 2017).

As BS-QUAL seems to indicate, the key internal factors that business school managers must pay special attention to include the following variables: variables related to teachers, especially those that give security to the student; the accreditation and experience of the teacher; and variables that make the student feel supported and that the teacher is powerful at the communicative level and is available to the student. In addition to these factors, the physical conditions and environment in which teaching and learning occur are also key to satisfying the student.

Regarding the second large group of variables that are part of the designed scale, the so-called external factors, they are grouped into four dimensions: sustainability, internationalization and external relations, notoriety, and preparation for the future. They are classified as external factors to these dimensions because they affect variables that analyse how the business school is related to its environment. Previous studies (McMahon, 2012; Mazzarol, 1998) confirm that interactions with stakeholders as part of higher education’s environment encompass the quality of service. The “core” of external factors to be controlled to maximize the satisfaction perceived by the student are the position the organization takes in terms of sustainability, ethics, or environmental issues. These issues include how the school interacts with other business schools and how it is positioned in rankings and the media or how it prepares its graduates for the labour market.

It is noteworthy, in terms of the dimensions that refer to external factors, that two hybrid dimensions were created: “Internationalization and Relations” and “Notoriety”. These two new dimensions are the result of the fusion of variables from three of the initial dimensions detailed in the literature. These are “Image”, “Internationalization”, and “Knowledge transfer” (Alves & Raposo, 2007; Gibson (2010); Urgel, 2007; Jewett, 2012; Dyllick, 2015; Lagrosen, 2017). The resulting grouping into only two final dimensions is logical.

Regarding the new dimension “Notoriety”, the responses to the survey seem to indicate that business school graduates consider the impact of a particular initiative to be much more important than how this impact was achieved. For example, it seems that having positive impacts in the press and social networks to gain notoriety is more important than the reason for appearing in these media, whether due to research, a sector study, a prominent position in rankings, an interview with a professor in a prestigious newspaper, or the creation of a business professorship. Lejeune et al. (2019) also considered citations of papers published in leading journals and positioning in business press rankings as two impact measures of business schools. That is, the positive impact and the increase in the presence of the business school in the market are more important than the impact achieved. In this sense, for the hybrid dimension “Notoriety”, related variables of the initial dimensions “Image” (Srikatanyoo & Gnoth, 2012) and “Knowledge transfer” appear. Our research seems to indicate that perceived quality is strongly influenced by the presence and notoriety that a business school has in the market and the environment in which the student develops. The presence and notoriety of a university are suggested to be part of the university’s reputation by some authors (Krampf & Heinlein, 2014; Price et al., 2013).

The second hybrid dimension generated by the scale refers to how the business school is related to other schools and the relationships it has with other prestigious business schools. In this case, variables of the initial dimension “Image” with the greatest impact on the visibility of a business school outside its country of origin are combined with some variables of the initial dimension “Internationalization”. This final grouping seems to indicate that the student of a business school values and perceives as high quality business schools that are well valued internationally and that facilitate student exchanges with other organizations of similar or greater prestige. These statements follow previous research (Alves & Raposo, 2007; Urgel, 2007; Jewett, 2012). The senior management of business schools should not only take care of the internal image of their institutions but also promote win–win relationships with prestigious entities. Part of these relations are with the stakeholders with whom the university operates, as these relations are considered important. This item, perceived as indicating quality by the student, does not have to assume a monetary cost for the business school since it can be materialized mainly through agreements.

Two initial dimensions related to the aforementioned external factors remain intact in the scale; these are the “Sustainability” and “Preparation for the future” dimensions (Gibson, 2010; Leblanc & Nguyen, 1999; Mai., 2005). Regarding “sustainability”, all the data seem to indicate that a student likes to study at an organization committed to ethics, the environment, and gender equality (Snelson-Powell et al., 2016). In line with what has been provided by previous literature (Gibson, 2010; Lagrosen, 2017; Leblanc & Nguyen, 1999), the existence of clear and well-communicated sustainability policies is an important part of the value generation of business schools and is an important component of the quality perceived by students. This result sheds light on the nascent stages of sustainability in business schools stated by Gupta and Singhal (2017). Dimensions of sustainability include social activities with the community, which is in line with Sokoli et al. (2018), who identified the social interest of students as part of their satisfaction with an institution.

Not surprising and consistent with previous comments is the presence in the scale of the dimension “Preparation for the future”, composed of variables clearly related to the quality of jobs of graduates (Gault et al., 2010; Gibson, 2010; Letcher & Neves, 2010; Mai, 2005). The variables that make up this dimension are highly valued by the most famous quality accreditations of business schools (AMBA, EQUIS, and AACSB).

To conclude, an analysis of the results shows that of the seven variables added to the survey by business school managers, four were kept in the scale. In this sense, the use of a mixed analysis method to enrich the survey was useful. Specifically, two variables are part of the final dimension of “Sustainability”: “Notoriety” and “Preparation for the future”. The four expanded dimensions are made up of external factors that influence the student’s perception of quality.

At the global level, everything seems to indicate that those who choose to go to business school consider the amount paid as an investment, which not only generates benefits in personal preparation and content (internal dimensions) but also seeks returns related to the improvement of personal brand/image/prestige and to personal growth and better job positioning (external dimensions).

## Conclusions

The main contribution of this article is the design of a scale that captures the perceptions of the student throughout his “academic journey”, not only internally and during business school but also at the levels of external and environmental factors that determine student satisfaction with the business school in line with the TPB framework. In this sense, the correct use of this scale can help the top management of business schools establish strategies that minimize the distance between student expectations and services provided and coveted academic excellence.

The scale also incorporates benefits in the sense that it not only is it a product of the previous literature and subsequent validation of the students but also has been enriched with the know-how and experience of current senior business school managers. The use of mixed methods has enriched the scale by incorporating opinions and the valuation of the three main agents that should intervene in an exploratory study. These agents are researchers who have previously analysed the object of study (previous literature), the clients who receive the service (students), and those providing the service (business schools, and more specifically, the top managers of these institutions). For all the contributions mentioned, we believe that this article adds value to the literature and can help both researchers and practitioners.

We must not forget that this paper is subject to limitations derived from its own nature. As this is an exploratory study, it is not intended to set laws but to lay the foundations for future research. Another limitation to take into account has to do with the survey employed, which was conducted at the national level. In this sense, the present study does not intend to generalize, and the authors accept that in different countries, cultures, or other normative-legal environments, the conclusions obtained may vary.

Furthermore, some future lines of research are proposed. First, the present scale can be adapted to different situations and contexts and, if necessary, supplemented with additional dimensions not currently included in the scale. Second, there is the possibility of analysing whether high degrees of quality perceived by students imply corresponding positions of business schools in the most prestigious international rankings. Third, the professor’s position could be of interest for understanding student satisfaction, as Owlia and Aspinwall (1996) pointed out. In this line, it will be interesting to measure differences in student satisfaction controlled by professors’ positions. Finally, this exploratory study serves as a first step to designing a global scale suitable for business schools independent of other contextual factors, such as the particular higher education system.

## Appendix 1

Table 7

Table 8

**Table 7:** Items proposed for the scale grouped by dimension based on the literature review

Original Code		Item	Original dimension	Mean	Standard deviation	References
1	AS1	Academic and professional accreditations for teachers	Academic staff	4	0.94	Borden (1995) Browne et al. (1998) Delaney (2001) DeShields et al. (2005) Elliot and Shin (2002) Elliot (2002–03) Leblanc and Nguyen (1999) Mai (2005) Tsinidou et al. (2010) Gibson (2010)
2	AS2	Experience and interest of the teacher in the area of knowledge taught		4.6	0.52	
3	AS3	The teacher’s closeness to the students		4.6	0.52	
4	AS4	Availability of the teacher (tutorials, etc.)		3.8	0.79	
5	AS5	Communication skills of teachers		4.9	0.32	
6	AS6	The teacher is innovative and acts as an agent of change		4.1	0.57	
7	AS7	Quality and productivity of the teacher as a researcher		3.1	0.99	
8	CV1	General design and methodology adapted to the participant (face-to-face, blended, online, etc.)	Classes/CV	4.6	0.52	Borden (1995) Browne et al. (1998) Delaney (2001) DeShields et al. (2005) Elliot and Shin (2002) Elliot (2002–03) Gibson (2010) Leblanc and Nguyen (1997) Mai (2005) Tsinidou et al. (2010) Letcher and Neves (2010)
9	CV2	Usefulness of the course in the personal and professional life of students		4.6	0.70	
10	CV3	Compatibility of schedules with personal-work life		4.0	0.82	
11	CV4	Appropriate number of persons in classroom		3.9	0.88	
12	CV5	Typology and logistics of classrooms		3.3	0.95	
13	ADV1	Accessibility and friendliness of the staff	Advising support	3.5	0.71	Borden (1995) DeShields et al. (2005) Elliot and Shin (2002) Gibson (2010)
14	ADV2	Clarity and usefulness of the information provided by the staff		3.5	0.71	
15	ADV3	Sensitivity to the concerns or suggestions of the students		3.9	0.74	
16	ADV4	Rapid responsiveness of staff		3.7	0.82	
17	SK1	Relational skills	Skills Developed	3.6	1.07	Browne et al. (1998) DeShields et al. (2005) Marimon et al. (2019) Gibson (2010) Leblanc and Nguyen (1999)
18	SK2	Critical thinking		3.7	0.67	
19	SK3	Intellectual growth		3.7	0.67	
20	SK4	Social/moral awareness		4.1	0.57	
21	SK5	Environmental awareness		4.1	0.88	
22	SERV1	Availability of parking	Service and Facilities	2.7	1.25	Borden (1995) Delaney (2001) Elliot and Shin (2002) Pariseau and McDaniel (1997) Gibson (2010) Leblanc and Nguyen (1999) Mai (2005) Marimon et al. (2019) Tsinidou et al. (2010)
23	SERV2	Good connections with public transport		3.9	0.74	
24	SERV3	Availability of computer classrooms		2.4	1.07	
25	SERV4	Availability of IT support personnel		2.7	1.34	
26	SERV5	Service of study classrooms		3.4	1.07	
27	SERV6	Library Service		2.5	0.85	
28	SERV7	Availability of scholarships and financial aid		3.8	0.79	
29	SERV8	Adequacy of quality/price of the programmes		4.4	0.70	
30	SERV9	Location of the building (proximity to the centre, nearby utilities…)		3.8	1.14	
31	SERV10	Decoration, environment, and general cleanliness		3.7	0.95	
32	SERV11	Campus security		3.5	0.71	
33	SERV12	Availability of internal catering services		3.1	0.99	
34	SUST1	Opportunities to socialize	Sustainability	3.5	1.08	Borden (1995) Elliot and Shin (2002) Delaney (2001) Elliot (2002–03) Tsinidou et al. (2010) Urgel (2007) Dyllick (2015) Leblanc and Nguyen (1999) Gibson (2010) Lagrosen (2017)
35	SUST2	The centre is committed to gender equality policies		3.5	0.85	
36	SUST3	The centre has a clear accreditation/sustainability policy		3.7	0.82	
37	SUST4	Interculturality of students/teachers/staff in the centre		3.8	0.63	
38	**SUST5***	Environmental commitment of the centre		3.5	0.53	
39	SUST6	Contribution of the institution to society		3.9	0.74	
40	SUST7	Commitment to sustainability (ethics, gender, environment…)		3.9	0.88	
41	SUST8	Social activities (collaboration with NGOs, development projects, etc.)		3.3	0.48	
42	IMG1	Brand awareness	Image	4.5	0.53	Urgel (2007) Jewett (2012) Alves and Raposo (2007)
43	IMG2	Being part of an international network of business schools		4	0.82	
44	IMG3	Availability of national accreditations		4.3	0.82	
45	IMG4	Availability of international accreditations		4.2	0.79	
46	IMG5	Presence in national and international rankings		4.5	0.85	
47	IMG6	Increasing presence in the institution’s market (increase in market share)		4	0.82	
48	IMG7	Accreditations of the business school (EQUIS, AMBA…)		4.4	0.70	
49	IMG8	Prestige and visibility of the business school		4.7	0.48	
50	IMG9	Others speak positively of the institution		4.7	0.48	
51	KT1	Number of Professorships	Knowledge Transfer	3	0.94	Urgel (2007)
52	KT2	Number of patents		2.5	0.71	
53	**KT3***	EUR invested in R&D projects		2.7	0.82	
54	KT4	Consulting projects and other business relationships		3.6	0.84	
55	KT5	Impact on the press		4.5	0.53	
56	**KT6***	Impact on social networks		4.5	0.53	
57	**KT7***	Organization of professional events or impact research		3.6	0.97	
58	INT1	The agreements with other universities are of quality	Internationalization	4	1.05	Urgel (2007) Jewett (2012) Alves and Raposo (2007)
59	INT2	The institution has agreements that allow students to pursue credits in other relevant business schools		4.2	0.63	
60	INT3	Mobility of teachers		3	0.94	
61	INT4	Mobility of administrative and service staff		2	1.05	
62	INT5	Participation in international projects		2.9	0.88	
63	INN1	The courses use innovative learning methods	Innovation	4	0.67	Gibson (2010) Lagrosen (2017) Dyllick (2015)
64	INN2	The contents of the courses are conveniently updated		4.5	0.53	
65	INN3	The institution promotes innovation, and there is a clear culture of innovation		4.1	0.74	
66	**INN4***	The institution is up to date with the latest news, applications, and content regarding online platforms		4.3	0.67	
67	**FUT1***	% of employed persons one year after graduating	Preparation to the future	4.6	0.52	Borden (1995) Debnath et al. (2005) DeShields et al. (2005) Gibson (2010) Letcher and Neves (2010) Gault et al. (2010) Mai (2005) Leblanc and Nguyen (1999)
68	**FUT2***	% salary increase two years after graduating		4.4	0.70	
69	FUT3	Increase in the network of professional contacts (companies, alumni,…)		4.5	0.71	
70	FUT4	Possibility of doing internships in companies while in school		4.4	0.84	
71	FUT5	Access to an efficient and competitive job bank		4.6	0.70	

## Appendix 2

**Table 8:** Scale

Final Dimension		Original code	Definitive code	Descriptive
Academic Staff	1	AS1	AS1	Academic and professional accreditations for teachers
	2	AS2	AS2	Experience and interest of the teacher in the area of knowledge taught
	3	AS4	AS3	Availability of the teacher (tutorials, etc.)
	4	AS5	AS4	Communication skills of teachers
Service and Facilities	5	SERV2	SERV1	Good connections with public transport
	6	SERV9	SERV2	Location of the building (proximity to the centre, nearby utilities…)
	7	SERV10	SERV3	Decoration, environment, and general cleanliness
	8	SERV11	SERV4	Campus security
Sustainability	9	SUST2	SUST1	The centre is committed to gender equality policies
	10	SUST3	SUST2	The centre has a clear accreditation/sustainability policy
	11	SUST5	SUST3	Environmental commitment of the centre
	12	SUST6	SUST4	Contribution of the institution to society
Internationalisation and Relations	13	IMG2	REL1	Being part of an international network of business schools
	14	IMG4	REL2	Availability of international accreditations
	15	INT1	REL3	The agreements with other universities are of quality
	16	INT2	REL4	The institution has agreements that allow students to pursue credits in other relevant business schools
Notoriety	17	KT5	NOT1	Impact on the press
	18	KT6	NOT2	Impact on social networks
	19	IMG5	NOT3	Presence in national and international rankings
	20	IMG6	NOT4	Increasing presence in the institution’s market (increase in market share)
Preparation to the future	21	FUT1	FUT1	% of employed persons one year after graduating
	22	FUT2	FUT2	% salary increase two years after graduating
	23	FUT5	FUT3	Access to an efficient and competitive job bank
